# Prevalence of Visual Impairment and Associated Factors among Adult Glaucoma Patients Attending Tertiary Eye Care Center in Gondar, Ethiopia, 2022

**DOI:** 10.4314/ejhs.v34i6.11

**Published:** 2024-11

**Authors:** Getenet Shumet Birhan, Gizachew Tilahun Belete, Fisseha Admassu Ayele, Biruk Lelisa Eticha

**Affiliations:** 1 Department of Optometry, College of Medicine and Health Sciences, Comprehensive Specialized Hospital, University of Gondar, Gondar, Ethiopia; 2 Department of Ophthalmology, College of Medicine and Health Sciences, Comprehensive Specialized Hospital, University of Gondar, Gondar, Ethiopia

**Keywords:** Visual impairment, Glaucoma, Adult, Ethiopia, Gondar

## Abstract

**Background:**

Glaucoma is a group of eye diseases characterized by damage to the optic nerve, often associated with increased intraocular pressure. Untreated damage from glaucoma can cause irreversible vision loss, making it one of the leading global causes of blindness. This study aimed to determine the prevalence of visual impairment and associated factors in adult glaucoma patients.

**Methods:**

An institutional cross-sectional study was conducted on a sample of 423 glaucoma patients selected by systematic random sampling from May 15 to June 30, 2022, at the University Gondar, Tertiary Eye Care and Training Center. Data were collected through personal interviews using a pre-tested structured questionnaire and a review of medical records. Descriptive statistics were summarized by frequency, percentage, and summary statistics. Binary logistic regression was performed, and variables with a P value <0.05 in the multivariable model were considered significantly associated.

**Results:**

This study found visual impairment in 77.6% (95% CI: 74.2%–82.9%) of adult participants with glaucoma. Female gender (AOR=2.45, 95% CI=1.18–3.48), rural residence (AOR=2.45, 95% CI=1.91-3.33), primary open-angle glaucoma (AOR=1.83, 95% CI=1.35-2.97), advanced-stage glaucoma (AOR=2.54, 95% CI=1.05–6.17), and longer duration of diagnosis (AOR=3.89, 95% CI=2.29–6.58) were significantly associated with visual impairment in adult glaucoma patients.

**Conclusion:**

The results of this study showed that visual impairment was significantly higher in adult glaucoma patients. Female gender, rural residence, primary open-angle glaucoma, advanced stage glaucoma, and longer duration of diagnosis were significantly associated with visual impairment in adult glaucoma patients.

## Introduction

Glaucoma is a disease that damages the optic nerve of the eye by increasing the pressure caused by fluid buildup in the front of the eye (1). This damage can lead to visual impairment and blindness (2). Early detection and treatment are crucial to prevent visual impairment and minimize the economic burden of glaucoma in society (3).

The two most common types, primary open-angle glaucoma and primary angle-closure glaucoma, affect more than 2 million Americans and are becoming increasingly common (4). Many patients are asymptomatic at onset, leading to delayed diagnosis and treatment. This can have a significant impact on lifestyle and quality of life (5, 6). High intraocular pressure, advanced age, African descent, and vascular problems are known risk factors for glaucoma. There is growing evidence that vascular factors, including systemic problems such as high blood pressure and diabetes, as well as ocular factors such as blood flow and perfusion pressure, may also contribute to the development of glaucoma (7). The treatment of glaucoma consists of topical or systemic intraocular pressure-lowering agents, laser treatment or surgery (8).

According to the World Health Organization (WHO), glaucoma is responsible for 8% of all blindness worldwide, making it the second most common preventable cause of blindness after cataracts (9). In terms of regional differences, it is estimated that the prevalence of distance vision impairment is four times higher in low and middle-income countries than in high-income countries (2). In Thailand, 23.8% of patients diagnosed with glaucoma were found to have glaucomatous blindness (10). Another study among Egyptian glaucoma patients revealed that 5% were legally blind due to the condition, with a high likelihood of total blindness in affected eyes. Interventional strategies are recommended to address visual disability in Egyptian glaucoma patients (11). According to national surveys, glaucoma is the second most common cause of blindness in Nigeria following cataract (12), and it was the fifth leading cause of blindness in Ethiopia, accounting for 5.2% of all cases of blindness (13).

Visual impairment caused by glaucoma has numerous consequences for patients' daily lives, which in turn affect their quality of life. Glaucoma-related visual impairment should be prevented, although the burden of functional visual impairment due to this disease has not yet been fully recognized (14). Low best-corrected visual acuity has been shown to be associated with reduced ability to perform daily activities, increased risk of car accidents and falls, and increased mortality (10, 15). In addition, peripheral vision loss is associated with an increased risk of falls, car accidents and serious injuries.

Blindness due to glaucoma has been associated with an advanced stage of glaucoma at diagnosis, fluctuations in intraocular pressure during treatment, the presence of exfoliation syndrome and poor patient compliance (16). In addition, the incidence of glaucomatous blindness is higher in men than in women and typically increases with age (17).

Despite the high burden of glaucoma in Africa, including Ethiopia, there is limited information on the prevalence of visual impairment (VI) and associated factors among Ethiopian glaucoma patients. Studying this is crucial for developing effective interventions, shaping public health policies, enhancing early detection and treatment, and reducing blindness. Therefore, this study investigated visual impairment and associated factors among adult glaucoma patients in Ethiopia.

## Materials and Methods

**Study area and period**: This cross-sectional study took place at the University of Gondar, Comprehensive Specialized Hospital, Tertiary Eye Care and Training Center (UoGCSH-TECTC) from May 15^th^ to June 30^th^, 2022. The center is located in Gondar city, approximately 750 kilometers northwest of Addis Ababa, the capital city of Ethiopia. It provides medical therapy, surgical therapy, laser therapy, and refraction with optical correction for 31,200 patients annually. Additionally, it serves as a center for training and research (18). Glaucoma patients were diagnosed and managed five days a week before they could be followed up three days a week on Monday, Wednesday, and Friday.

**Sample size calculation**: Due to the absence of similar study conducted in comparable study population and setting, we have obligued to use single population proportion formula, taking the account of 50% proportion of expected visual impairment, 5% level of significance with a 95% confidence level , 0.05 margin of error, and 95% confidence interval. The sample size calculated was 384. After adding a 10% nonresponse rate, the final sample size was found to be 423.

**Sampling technique and procedure**: A systematic random sampling method was used to select the participants. We excluded participants who had hearing and speaking problems or a history of psychosocial disorders that would affect their ability to respond to the questionnaire. Based on the available data, the center sees an average of 150 glaucoma patients per week, about 50 per day, and 900 per month over two weeks. The sampling fraction was 2 (900 divided by 423). The first patient was randomly chosen using a lottery from numbers 1 to 2, and subsequent participants were selected by adding the sampling fraction to the number of the first chosen person. Each day, 24 patients were selected, with 15 selected on the final day of data collection. To achieve the desired sample size within the specified time period, if the number of patients attending the clinic per day was 24 or fewer, all of them were considered as study participants, provided they met the inclusion criteria without considering the sampling fraction.

### Operational definition

#### Visual impairment

**Distance VI**: Presenting distance visual acuity of the better eye worse than 6/12 (19)

**Blind**: Presenting distance visual acuity of the better eye worse than 3/60 (19)

**Psychosocial disorder**: Individuals with a mental health condition interact with a social environment that presents barriers to their equality with others (20)

**Adult**: A person who is 18 years old or above (21)

**Data collection tool and procedures**: The data were collected by using the Amharic version of a pretested, structured questionnaire consisting of questions about the prevalence of VI and its associated factors. Face-to-face interviews to estimate VI and patient medical chart reviews using a checklist to determine clinical factors were performed by 4 trained BSc optometrists, and supervision was performed by two MSc optometrists and principal investigator. The diagnosis and classification of glaucoma subtypes were confirmed through comprehensive clinical evaluations conducted by glaucoma specialists. These evaluations included detailed history taking, slit-lamp examination, intraocular pressure (IOP) measurement using Goldmann applanation tonometry, gonioscopy, visual field testing (automated perimetry), and optical coherence tomography (OCT) of the optic nerve head and retinal nerve fiber layer. Glaucoma subtypes such as primary open-angle glaucoma (POAG), primary angle-closure glaucoma (PACG), secondary open-angle glaucoma (SOAG), secondary angle-closure glaucoma, and ocular hypertension were classified according to established diagnostic criteria.

**Data processing and analysis**: The data were entered into epi info version 7 and exported to, checked, cleaned, and analyzed by using Statistical Package for Social Sciences (SPSS) version 26. The analysis was performed by the investigator using the same statistical package. Descriptive statistics such as proportions, frequencies, ratios, and summary statistics were calculated.

Binary logistic regression was performed to determine factors associated with visual impairment. All variables with a p value less than 0.2 were entered into the multivariable logistic regression for analysis. Variables with p value less than 0.05 in the multivariable logistic regression analysis were considered to be significantly associated. The adjusted odds ratio (AOR) with 95% confidence interval (CI) was used to show the strength of the association.

**Data quality assurance**: The first step involved translating the questionnaire to the local language Amharic and retranslating it back to language experts. All the data collectors were trained for two days before the data collection process, and the questionnaires were pretested to check for completeness, appropriateness and common understanding. To ensure the quality of the data, the principal investigator closely supervised the data collection process daily. A review was made in the field, and the data were codded for data entry.

## Results

**Sociodemographic and economic characteristics of the study participants**: A total of 415 participants completed the study, resulting in a response rate of 98.1%. The average age of the participants was 56.53 years, with a standard deviation of 12.08 years. Out of the total participants, 305 (73.5%) were females, 313 (75.4%) were married, 120 (28.9%) were unable to read and write, 157 (37.8%) were farmers, and 234 (56.4%) were urban residents. The median monthly family income of the respondents was 2000 Ethiopian birrs (ETB) with an interquartile range (IQR) of 3000 Ethiopian birrs (Table 1).

**Table 1:** Sociodemographic characteristics of adult glaucoma patients enrolled in the study (n=415)

Variables	Category	Frequency	Percent
Age in years			
	18-40	42	10.1%
	41-60	231	55.7%
	≥61	142	34.2%
Sex			
	Male	110	26.5%
	Female	305	73.5%
Residence			
	Urban	234	56.4%
	Rural	181	43.6%
Religion			
	Christian	350	84.3%
	Muslim	65	15.7%
Marital status			
	Single	24	5.8%
	Married	313	75.4%
	Divorced	36	8.7%
	Widowed	42	10.1%
Educational status			
	Can't read and write	120	28.9%
	Read and write only	150	36.1%
	Primary education	37	8.9%
	Secondary education	53	12.8%
	Collage and above	55	13.3%
Occupation			
	Farmer	157	37.8%
	Self employed	52	12.6%
	Civil servant	44	10.6%
	Merchant	64	15.4%
	Retired	98	23.6%
Average family monthly income (in Ethiopian Birr)			
	≤ 1000	114	27.5%
	1001-1900	81	19.5%
	1901-4000	126	30.4%
	>400	94	22.6%
Family history of glaucoma			
	Yes	81	19.5%
	No	334	80.5%

n: Sample size

**Clinical characteristics of the study participants**: In terms of ocular clinical characteristics, the most prevalent type of glaucoma was primary open-angle glaucoma 236 (56.9%). A majority of the participants had moderate glaucoma 166 (40%), had been diagnosed for more than twelve months 269 (64.8%), and had glaucoma in both eyes 290 (69.9%). Additionally, the majority of patients were receiving drug treatment 348 (83.9%) (Table 2).

**Table 2:** Clinical characteristics of adult glaucoma patients participated in the study (n=415)

Variables	Category	Frequency	Percent
IOP in mmHg			
	<21	231	55.7%
	21-30	138	33.3%
	>30	46	11.0%
Cup to disc ratio			
	<0.65	67	16.1%
	0.7–0.9	261	62.9%
	>0.9	87	21.0%
Type of glaucoma			
	POAG	236	56.9%
	Angle closure glaucoma	78	18.8%
	Pseudoexfoliative glaucoma	69	16.6%
	SOAG	32	7.7%
Severity of glaucoma			
	Mild	69	16.6%
	Moderate	166	40.0%
	Advanced	123	29.7%
	Absolute	57	13.7%
Laterality of glaucoma			
	Bilateral	316	76.1%
	Unilateral	99	23.9%
Type of treatment			
	Drug only	348	83.9%
	Drug and surgery	67	16.1%
Duration of diagnosis			
	≤ 12 month	125	30.1%
	> 12 month	290	69.9%
History of eye surgery			
	Yes	133	32.0%
	No	282	68.0%
Chronic systemic illness			
	Yes	113	30.1%
	No	302	69.9%

Note- n: Sample size; IOP: Intraocular Pressure; mmHg: Millimeter of mercury; POAG: Primary Open Angle Glaucoma; SOAG: Secondary Open Angle Glaucoma; SOAG includes inflammatory glaucoma, phacolytic glaucoma, and pigment dispersion glaucoma; Chronic systemic illness includes diabetes mellitus, hypertension, and cardiovascular disease

**Prevalence of visual impairment**: In the present study, the prevalence of visual impairment among glaucoma patients was 77.6 %( 95% CI: 74.2%-82.9%). Among 328 visually impaired glaucoma patients, 145(34.9%) had moderate visual impairment, and 135 (32.5%) had blindness.

**Factors associated with visual impairment**: The variables were separately entered into a bivariable logistic regression model. Sex, residence, religion, occupation, IOP, cup-to-disc ratio, type of glaucoma, severity of glaucoma, laterality of glaucoma, type of glaucoma treatment, duration of diagnosis, history of eye surgery, and chronic medical illness, which had p values less than 0.2 and were subsequently fitted to a multivariable logistic regression. Finally, a significant association was determined by the enter method in the multivariable logistic regression, and a P value < 0.05 was considered to indicate a significant association with VI among glaucoma patients.

In this study, female sex, rural residence, primary open angle glaucoma, advanced stage glaucoma and longer duration of diagnosis were found to be significantly associated with VI among glaucoma patients.

The likelihood of VI was approximately twofold greater in women than in men (AOR=2.45, 95% CI=1.18-3.48). Compared with participants residing in urban areas, those living in rural areas were nearly two and half times more likely to become visually impaired (AOR=2.45 95% CI=1.91-3.33).

Study participants with a duration of diagnosis greater than twelve months were nearly four times more likely to be visually impaired than participants with a duration of diagnosis <12 months (AOR=3.89, 95% CI= 2.29-6.58).

This study revealed that patients with primary open angle glaucoma (POAG) were nearly two times more likely to have VI than were those with secondary open angle glaucoma (SOAG) (AOR=1.83, 95% CI= 1.35-2.97). Similarly, study participants with absolute glaucoma were 2.54 (AOR=2.54, 95% CI=1.05-6.17) times more likely to be visually impaired than those with mild glaucoma (Table 3).

**Table 3:** Factors associated with visual impairment among adult glaucoma patients enrolled in the study (n=415)

Variable	Visual impairment		COR(95%CI)	AOR(95%CI)
			
	Yes (# and %)	No (# and %)		
Sex				
Female	168 (55.1%)	137 (44.9%)	2.23(1.37-3.36)	2.45(1.18-3.48)*
Male	40 (36.4%)	70 (63.6%)	1.00	1.00
Residence				
Rural	76(42.0%)	105(58.0%)	2.79(1.21-3.65)	2.45(1.91-3.33) *
Urban	132 (56.4%)	102 (43.6%)	1.00	1.00
Religion				
Christian	184 (52.6%)	166 (47.4%)	1.00	1.00
Muslim	24 (36.9%)	41 (63.1%)	0.53(0.31-0.91)	0.62(0.32-1.19)
Occupation				
Farmer	91 (58.0%)	66 (42.0%)	1	1
Self employed	25 (48.1%)	27 (51.9%)	0.67(0.36-1.26)	0.79(0.37-1.67)
Civil servant	20 (45.4%)	24 (54.6%)	0.60(0.31-1.18)	0.65(0.30-1.43)
Merchant	30 (46.9%)	34 (53.1%)	0.64(0.36-1.15)	0.61(0.30-1.23)
Retired	42 (42.8%)	56 (57.2%)	0.54(0.33-0.91)	0.59(0.32-1.08)
IOP in mmHg				
<21	125 (54.1%)	106 (45.9%)	1.00	1.00
21-30	57 (41.3%)	81 (58.7%)	0.60(0.39-1.91)	0.69(0.41-1.15)
>30	26 (56.5%)	20 (43.5%)	1.10(0.58-2.09)	1.12(0.52-2.40)
Cup to disc ratio				
≤0.65	32 (47.8%)	35 (52.2%)	1.00	1.00
0.7–0.9	132 (50.6%)	129 (49.4%)	1.12(0.65-1.91)	1.30(0.67-2.52)
>0.9	44 (50.6%)	43 (49.4%)	1.12(0.59-2.12)	1.10(0.52-2.34)
Type of glaucoma				
POAG	110(46.6%)	126 (53.4%)	1.99(1.47-2.07)	1.83(1.35-2.97) *
Angle closure glaucoma	43 (55.1%)	35 (44.9%)	1.39(0.61-3.18)	0.75(0.28-2.02)
Pseudoexfoliative glaucoma	40 (57.9%)	29 (42.1%)	1.56(0.67-3.63)	1.57(0.59-4.17)
SOAG	15 (46.9%)	17 (53.1%)	1.00	1.00
Severity of glaucoma				
Mild	31 (44.9%)	38 (55.1%)	1.00	1.00
Moderate	83 (50.0%)	83 (50.0%)	1.23(0.70-2.15)	1.32(0.67-2.62)
Advanced	65 (52.8%)	58 (47.2%)	1.37(0.76-2.48)	1.88(0.91-3.91)
Absolute	29 (50.9%)	28 (49.1%)	1.27(0.63-2.56)	2.54(1.05-6.17)*
Laterality of glaucoma				
Unilateral	37 (37.4%)	62 (62.6%)	1.00	1.00
Bilateral	171 (54.1%)	145 (45.9%)	0.98(0.24-3.14)	0.32(0.33-4.06)
Type of treatment				
Drugs only	162 (46.5%)	186 (53.5%)	1.00	1.00
Drug and surgery	46 (68.6%)	21 (31.4%)	2.52(1.44-4.39)	1.89(1.00-3.58)
Duration of diagnosis				
< 12 month	34 (27.2%)	91 (72.8%)	1.00	1.00
> 12 month	174 (60.0%)	116 (40.0%)	4.02(2.54-6.35)	3.89(2.29-6.58)*
History of eye surgery				
Yes	75 (56.4%)	58 (43.6%)	1.45(0.96-2.19)	1.51(0.92-2.50)
No	133 (47.2%)	149 (52.8%)	1.00	1.00
Chronic medical illness				
Yes	70 (61.9%)	43 (38.1%)	0.93(0.24-3.01)	0.76(0.04-2.99)
No	138 (45.7%)	164 (54.3%)	1.00	1.00

Note- #: Number; ^*^: P value < 0.05; IOP: Intraocular Pressure; mmHg: Millimeter of mercury; POAG: Primary Open Angle Glaucoma; SOAG: Secondary Open Angle Glaucoma; SOAG includes inflammatory glaucoma, phacolytic glaucoma, and pigment dispersion glaucoma: Chronic systemic illness includes diabetes mellitus, hypertension, and cardiovascular disease

## Discussion

This cross-sectional study conducted at UoGCSH-TECTC assessed the prevalence of visual impairment and its associated factors among adult glaucoma patients. In the present study, the prevalence of visual impairment among glaucoma patients was 77.6% (95% CI: 74.2%-82.9%), which was notably higher compared to findings from studies conducted in Netherland (26%) (22), Ireland (45%) (23), Finland (16%) (16), and India (26%) (24). This discrepancy may be attributed to several factors, including socioeconomic differences, variations in the operational definitions of VI, methodological differences, and differences in study populations and sample sizes. For instance, the study from India employed visual field measurements as an additional criterion for assessing visual impairment, potentially leading to an underestimation of the true prevalence. Conversely, the study from Netherland focused on end-of-life visual impairment in patients monitored for glaucoma, which differs from the approach used in the present study which was assessed at a specific point in time. The other possible reason could be the study area in this research provides services for a large number of glaucoma patients and is the only tertiary eye care center in the region, which likely contributes to a higher number of visually impaired glaucoma patients.

In this study the prevalence of blindness among glaucoma patients was 32.5%, which was notably higher compared to findings from studies conducted in Thailand (23.8%) (10) and Egypt (5%) (11). This could be due to differences in healthcare access, awareness, and early detection. In Ethiopia, limited resources, fewer eye care professionals, and lower rates of screening contribute to late diagnosis and inadequate management of glaucoma, leading to higher rates of blindness. In addition, the UoGCSH-TECTC Glaucoma Clinic is the only referral center for northwestern Ethiopia, making it the last resort for referral of advanced cases, which could increase the rate of blindness. In contrast, Thailand and Egypt have more developed healthcare systems with better access to eye care services, regular screening programs, and public awareness initiatives. This output implicates that earlier detection and more health education regarding early eye care center visit is so much helpful.

In the present study, the likelihood of VI was about twice as high in women as in men, consistent with findings from a study done in USA (25). This disparity can be attributed to factors such as women's longer life expectancy (26), which increases the likelihood of age-related eye conditions, and a higher prevalence of diseases like cataracts and glaucoma. Additionally, socioeconomic factors, including reduced access to healthcare and educational disparities, contribute to delayed diagnosis and treatment in women. In many Ethiopian communities, men, as the primary breadwinners, have greater access to early screening. Biological differences, such as hormonal changes affecting eye health, also contribute to the higher incidence of visual impairment in women.

This study revealed that participants with rural residents were 2.45 times more likely to have VI compared to urban residents, consistent with findings from a study in Ghana (27). This may be due to a lack of health awareness and preventive measures for visual impairment, insufficient healthcare accessibility and infrastructure, long travel distances to eye clinics in rural areas, and unequal access to eye care services compared to urban areas. Additionally, lower living standards, educational levels, and poor environmental conditions and personal hygiene in rural areas contribute to a higher prevalence of eye infections and related visual impairment compared to urban areas (28). Rural residents often delay seeking medical attention for eye problems due to ignorance and lack of health awareness, typically seeking care only after vision impairment hinders their ability to work (28, 29).

The odds of being visually impaired were two times higher among participants with POAG than participants with SOAG (composed of inflammatory glaucoma, phacolytic glaucoma, and pigment dispersion glaucoma). This may be because POAG often progresses without noticeable symptoms until significant vision loss has occurred. This silent progression leads to a delayed diagnosis and treatment (30), allowing for more extensive optic nerve damage. In contrast, SOAG typically arises from identifiable causes such as trauma, inflammation, or other eye conditions, prompting earlier detection and intervention.

Patients with absolute-stage glaucoma were more than two times more likely to experience visual impairment than patients with mild glaucoma. This study finding was consistent with those of studies done in the USA (31, 32). This occurs because of the increase in retinal nerve fiber layer loss as the stage of glaucoma increases. Owing to the progressive nature of glaucoma and the permanence of visual impairment, early detection and intervention are vital for protecting the community from a highly compromised quality of life or for facilitating day-to-day activity.

The odds of developing visual impairment were approximately four times greater in glaucoma patients with a diagnosis duration exceeding twelve months compared to those with diagnosis duration of less than twelve months. This could be due to as disease chronicity increases; therapy-related, follow-up related and socioeconomic-related uncertainties can be encountered to facilitate damage to the optic nerve fiber layer. Additionally, the progressive nature of glaucoma could be a possible reason.

In conclusion, a significantly greater magnitude of visual impairment has been found among adult glaucoma patients. Female sex, rural residence, primary open angle glaucoma, advanced stage glaucoma, and longer duration of diagnosis were significantly associated with visual impairment among adult glaucoma patients.

This study has some limitations. Being cross-sectional, it does not assess changes in visual impairment over time or establish causal relationships between variables. Additionally, some data, such as visual field measurement, were not collected, and the small sample size may introduce confounding factors. Moreover, rather than performing gonioscopy for all glaucoma cases classification, it was performed for some of indicated patients such as patients who had a shallow anterior chamber angle, as identified by Van Herrick anterior chamber angle estimation technique, and for patients with risk factors for developing angle-closure glaucoma. To understand glaucoma-related visual disability and blindness in Ethiopia better, further multicenter studies across different regions are needed.
